# Optimising parallel R correlation matrix calculations on gene expression data using MapReduce

**DOI:** 10.1186/s12859-014-0351-9

**Published:** 2014-11-05

**Authors:** Shicai Wang, Ioannis Pandis, David Johnson, Ibrahim Emam, Florian Guitton, Axel Oehmichen, Yike Guo

**Affiliations:** Data Science Institute, Imperial College London, London, UK; School of Computer Science, Shanghai University, Shanghai, China

## Abstract

**Background:**

High-throughput molecular profiling data has been used to improve clinical decision making by stratifying subjects based on their molecular profiles. Unsupervised clustering algorithms can be used for stratification purposes. However, the current speed of the clustering algorithms cannot meet the requirement of large-scale molecular data due to poor performance of the correlation matrix calculation. With high-throughput sequencing technologies promising to produce even larger datasets per subject, we expect the performance of the state-of-the-art statistical algorithms to be further impacted unless efforts towards optimisation are carried out. MapReduce is a widely used high performance parallel framework that can solve the problem.

**Results:**

In this paper, we evaluate the current parallel modes for correlation calculation methods and introduce an efficient data distribution and parallel calculation algorithm based on MapReduce to optimise the correlation calculation. We studied the performance of our algorithm using two gene expression benchmarks. In the micro-benchmark, our implementation using MapReduce, based on the R package RHIPE, demonstrates a 3.26-5.83 fold increase compared to the default Snowfall and 1.56-1.64 fold increase compared to the basic RHIPE in the Euclidean, Pearson and Spearman correlations. Though vanilla R and the optimised Snowfall outperforms our optimised RHIPE in the micro-benchmark, they do not scale well with the macro-benchmark. In the macro-benchmark the optimised RHIPE performs 2.03-16.56 times faster than vanilla R. Benefiting from the 3.30-5.13 times faster data preparation, the optimised RHIPE performs 1.22-1.71 times faster than the optimised Snowfall. Both the optimised RHIPE and the optimised Snowfall successfully performs the Kendall correlation with TCGA dataset within 7 hours. Both of them conduct more than 30 times faster than the estimated vanilla R.

**Conclusions:**

The performance evaluation found that the new MapReduce algorithm and its implementation in RHIPE outperforms vanilla R and the conventional parallel algorithms implemented in R Snowfall. We propose that MapReduce framework holds great promise for large molecular data analysis, in particular for high-dimensional genomic data such as that demonstrated in the performance evaluation described in this paper. We aim to use this new algorithm as a basis for optimising high-throughput molecular data correlation calculation for Big Data.

## Background

Information from genomic, proteomic and metabolic measurements has already benefited identification of disease subgroups and the prediction of treatment responses of individual subjects, which is known as molecular profiling based patient stratification [1]. Biomedical research is moving towards using high-throughput molecular profiling data to improve clinical decision making. One approach for building classifiers is to stratify subjects based on their molecular profiles. Unsupervised clustering algorithms can be used for stratification purposes. This paper introduces significant optimisations to unsupervised clustering using four kinds of correlation methods with high-dimensional molecular profiling data (gene expression data), by taking full advantage of a programming model specifically designed for parallel processing of big datasets.

Our motivation for optimisation of unsupervised clustering is based on our experiences in using tranSMART, a biomedical data integration platform that includes support for clinical and molecular data [2]. TranSMART was originally developed by Johnson & Johnson for in-house clinical trial and knowledge management needs in translational studies. It has been open-sourced recently. Our aim is to optimise tranSMART so that clinicians can make use of it for faster and more confident clinical decision making. However, we have found that the performance of the R workflow currently used in tranSMART for preparing correlation matrices when analysing high-dimensional molecular data is sub-standard.

For example, we performed unsupervised hierarchical clustering on the publicly available Multiple myeloma (MULTMYEL) [3,4] dataset taken from NCBI’s Gene Expression Omnibus (GSE24080) [5,6]. The dataset contains 559 subjects’ gene expression data produced by an Affymetrix GeneChip Human Genome U133 Plus 2.0 Array. In order to build the classifiers, the subjects are clustered using a hierarchical clustering algorithm hclust() implemented in vanilla R [7]. In our preliminary tests, we found that running this algorithm on a virtual machine configured with 32 cores and 32 GB of memory took over 6 minutes calculating a Euclidean distance matrix using the function rdist() in package fields, and more than a week if performing a Kendall rank correlation using the function cor(). Based on these observations we concluded that the bottleneck in the hierarchical clustering algorithm was in generating the correlation matrix. Clearly optimising the performance of these analyses would expedite clinical decision making. With high-throughput sequencing technologies [8] promising to produce even larger datasets per subject, we expect the performance of the state-of-the-art statistical algorithms to be further impacted unless efforts towards optimisation are carried out.

In this paper our optimisation method applies on four kinds of correlation methods used to generate correlation matrices that are used by the hierarchical clustering algorithm in tranSMART – the Pearson product–moment correlation [9], Spearman’s rank-order correlation [10], Kendall’s rank correlation [11], and Euclidean distance correlation. We describe a series of processing optimisations, based around the MapReduce programming model [12], on the calculation of correlation matrices used for hierarchical clustering. We go on to present how our correlation matrix calculations implemented on R MapReduce package RHIPE [13], which works in combination with Hadoop [14], a robust and well-supported distributed data storage and computation framework that supports MapReduce, significantly outperforms their comparable implementations configured for distributed execution in R using Snowfall [15] package with Rmpi [16], a parallel computing package for R scripts.

## Methods

In data mining, hierarchical clustering is a method of cluster analysis that seeks to build a hierarchy of clusters using correlation matrices. Currently there are three main types of correlation coefficient calculation algorithms: Product–moment correlation, rank correlation and other dependence measurements. Pearson’s product–moment correlation is a measure of the linear correlation between two variables X and Y, giving a value between +1 and −1 inclusive, where 1 is total positive correlation, 0 is no correlation, and −1 is total negative correlation. Spearman and Kendall correlation methods are two examples of using a rank correlation coefficient. Spearman’s rank correlation coefficient is a non-parametric measure of statistical dependence between two variables by using a monotonic function. If there are no repeated data values, a perfect Spearman correlation of +1 or −1 occurs when each of the variables is a perfect monotone function of the other. Kendall tau rank correlation coefficient is a statistic used to measure the association between two measured quantities using a tau test, a non-parametric hypothesis test for statistical dependence based on the tau coefficient. Euclidean distance is a popular dependence measurement that differs from correlations by calculating the distance between two points according to Pythagorean theorem within a metric space.

R is a widely used analysis tool for clustering and correlation calculation. Many emerging parallel R packages, such as RHIPE, SparkR [17], RABID [18], Snowfall, Rmpi and pbdMPI [19], can be used to parallelize R processes. RHIPE is a Hadoop MapReduce based R package that transforms R functions into MapReduce jobs. SparkR and RABID are MapReduce packages, which works in combination with Apache Spark [20]. Though Hadoop performs slower than Spark in several cases, such as iterative computations, Hadoop is much more mature to provide a more stable performance. Snowfall with Rmpi is a combination of parallel packages that works in a master–slave interactive mode, where all code and data are distributed to each process within a cluster, then the code works respectively in each process, and finally the master collects the final result. While pbdMPI uses Single Program Multiple Data parallel programming model, which is not as popular as Snowfall.

MapReduce is a simple processing model based around three functions that execute at two distinct stages: the Mapper function, Map(), transforms raw input data into a set of intermediate key-value pairs; then the Combiner function, Combine(), sorts and packs the key-value pairs in the Map stage; finally the Reducer function, Reduce(), takes the related data (usually sorted by the key in the key-value pairs emitted by the Mapper) and merges the data together. The MapReduce task division is based around how the input data is distributed and processed by the Mapper and Reducer at each stage. Initially the input data is split and distributed to Mappers that run on different compute nodes. After emitting some key-value pairs, the MapReduce system sorts the pairs into related groups by key. These groups are then each provided as input to Reducers that also run on different compute nodes. Finally, the output of the Reducers in collected and combined into a single output dataset.

Our approach to applying MapReduce to calculating correlation matrices on gene expression data is inspired by work presented in Li Q, et. al. [21] who used a MapReduce-like model for distributing large datasets on GPUs to calculate Euclidean distance. In the case of our MapReduce implementation, our input corresponds to a set of vectors, each containing the probesets intensity values for a single subject. This input set of vectors is divided into data blocks that are dispatched to different Mappers, dependent on the total number of CPU cores available. In the Mapper stage, each data block is copied to each Reducer by emitting as a key the index of the corresponding Reducer and as the value the data block. Each Reducer then calculates correlation values by loading the data block corresponding to the key and calculating the correlation coefficients against each data block output from the Mappers.

**Figure 1:**
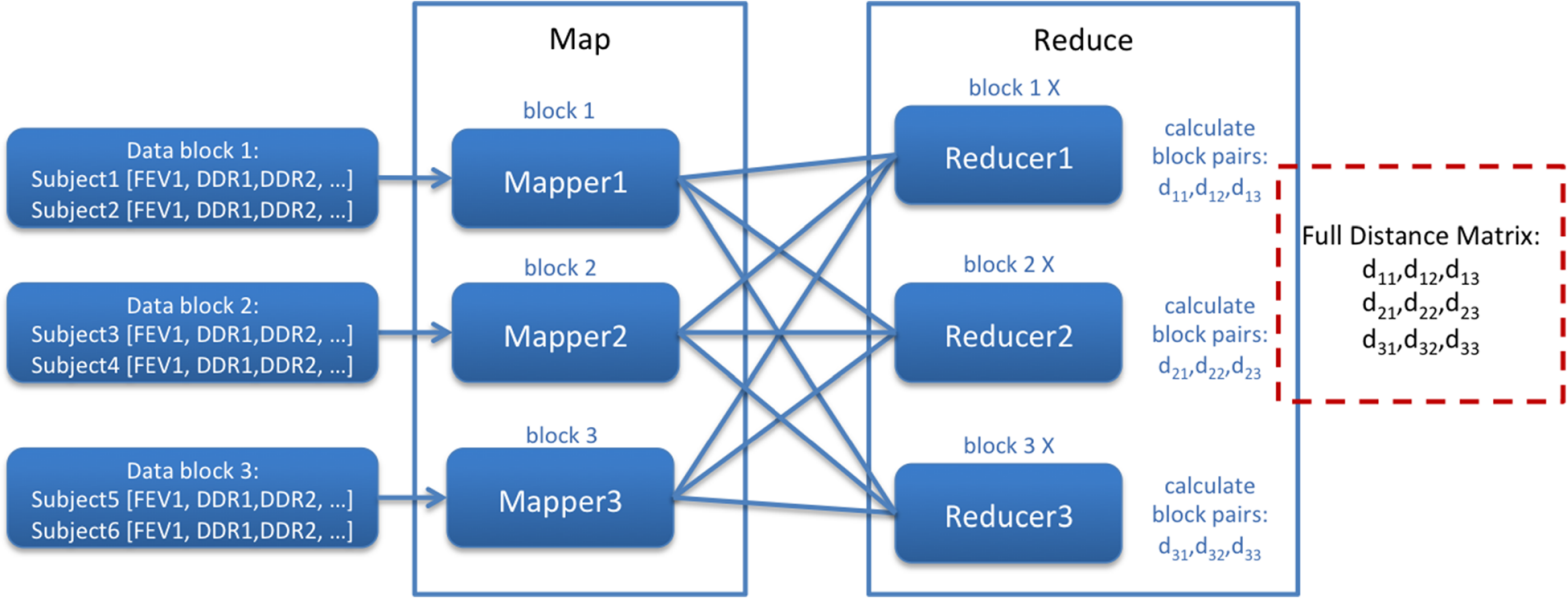
**Basic correlation matrix calculation using MapReduce.**

In the example shown in Figure 1, each data block can contain several subjects, where in this example we show two subjects per data block. Each block is loaded by a Mapper, and then copied to each Reducer. Based on the Reducer ID, a corresponding data block is loaded and coefficients calculated against all other data blocks. For example, Reducer1 loads Data block 1 and performs a pairwise comparison with outputs from each Mapper (Data block 1, Data block 2, and Data block 3), producing coefficient values d_11_, d_12_ and d_13_.

**Figure 2:**
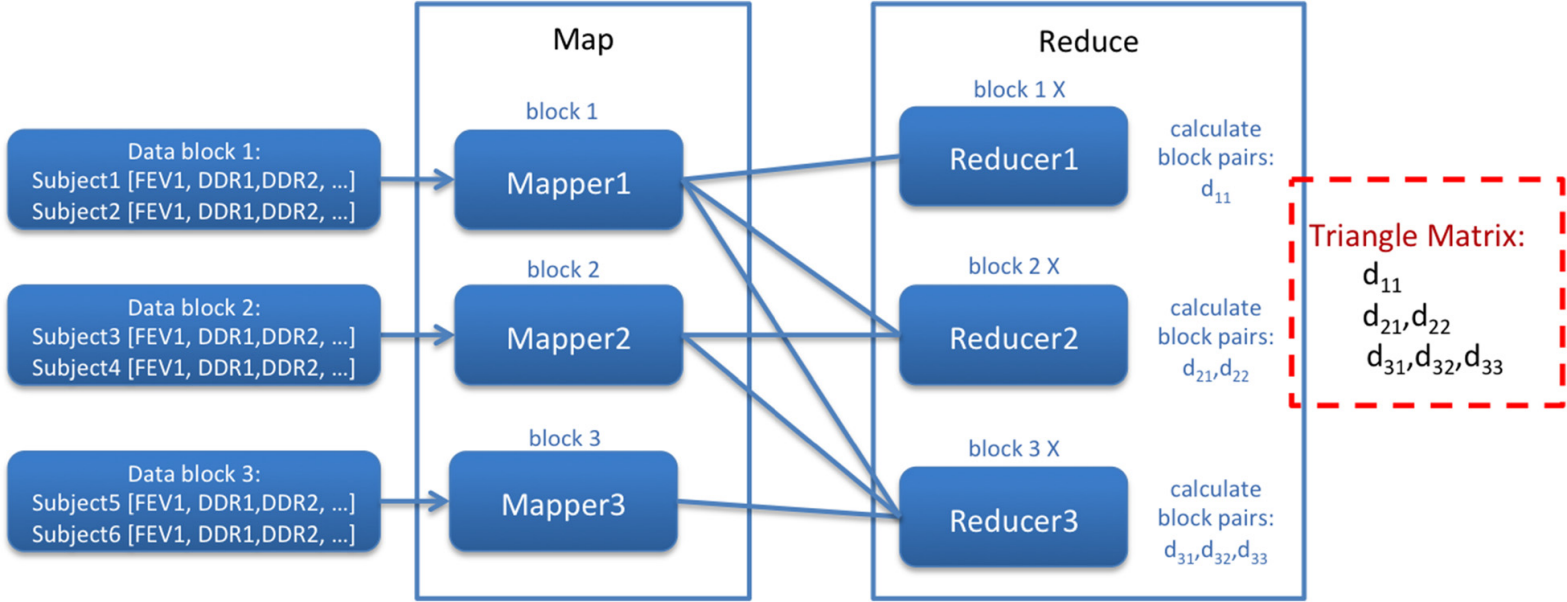
**Correlation matrix calculation with redundant coefficient calculations skipped.**

To optimise this MapReduce model, we made further modifications to the one presented by [21]. We note that there is a certain amount of redundancy in the calculation of a full correlation matrix. Each coefficient appears twice in the correlation matrix. Looking back at the example in Figure 1, we can see that each of d_12_ and d_21_, d_13_ and d_31_, and d_32_ and d_23_ correspond to the same pair-wise calculations. To avoid this, we can compare the ID of each Mapper with that of the target Reducer before distributing data. If the Data block ID is greater than the Reducer ID, then that particular data block distribution and correlation coefficient calculation can then be skipped. For example, in Figure 2 we can see that Mapper 2 does not send Data block 2 to Reducer 1. This results in only d_12_ being calculated, instead of both d_12_ and d_21_. This optimisation results in all correlation coefficients only being calculated once.

This optimisation the coefficient calculations shown in Figure 2 on results, however, now produces an imbalanced workload on the Reducers. If we look at Figure 2 more closely, we can see that Reducer 1 receives a workload corresponding to one pair-wise calculation (d_11_), while Reducer 2 pairs calculations (d_21_, d_22_), and so forth. With this pattern of workload, the longest running Reducer determines the overall performance of the MapReduce workflow. If we can balance the load on the Reducers, the workload will execute fully in parallel, thus reducing the overall execution time.

**Figure 3:**
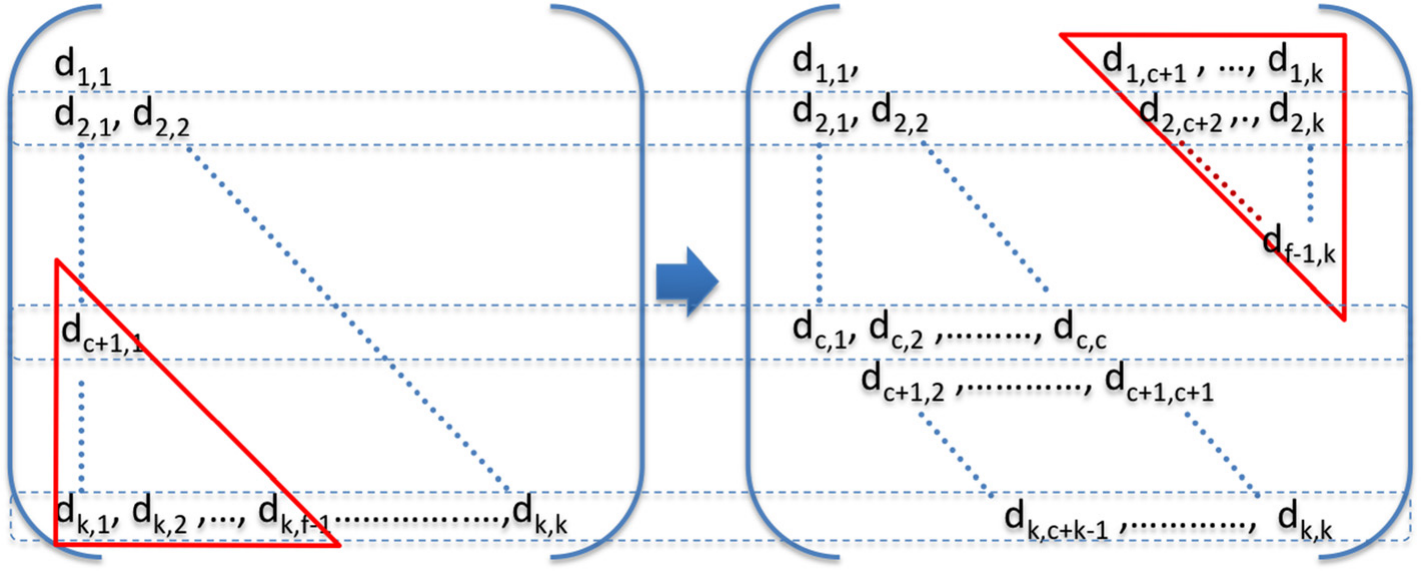
**Matrix transformation.** The elements in the bottom left triangle are mapped to the top right triangle. Each row represents a Reducer’s load, which contains either c elements or f elements.

To do this, we have used a matrix transformation algorithm (see pseudo code below) to balance all Reducers by moving the bottom left triangular section of the correlation matrix to the top right, as shown in Figure 3.

Denote:

- *k* is the number of all the data blocks.
- *i* is the id of *a* data block.
- *a* is the average number of distance calculation per Reducer, $a={\displaystyle \sum_{i=1}^k}i/k.$
- *c* is the ceiling of *a*, *c* = ⌈*a*⌉.
- *f* is the floor of *a*, *f* = ⌊*a*⌋.

Algorithm pseudo:

- For the data block *i* in each Mapper
- *if* (*i* < = *f*)
- Mapper send this block to Reducer_*i*_, Reducer_*i+1*_,…, Reducer_*i+f-1*_;
- *else if* (*i* > = *c*)
- Mapper send this block to Reducer_*i%k*_, Reducer_(*i+1*)*%k*_,…,Reducer_(*i+c-*1)%*k*_.

**Figure 4:**
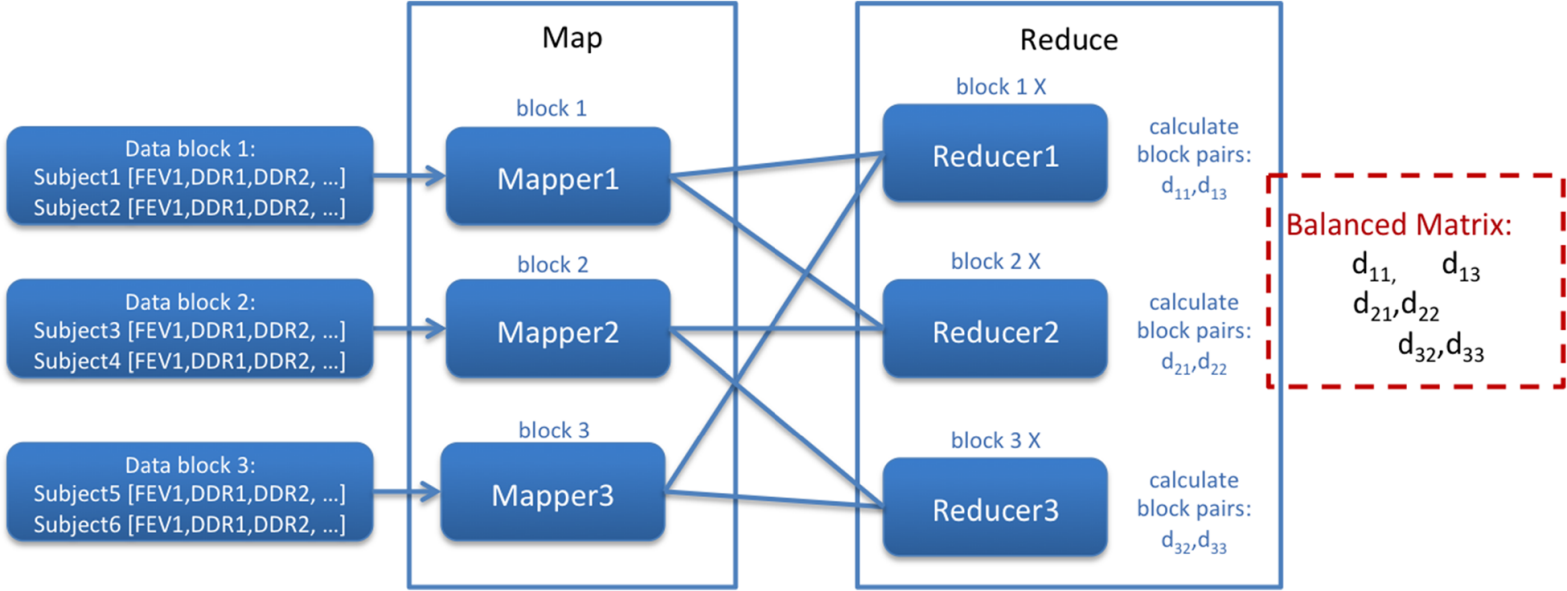
**Balanced reducers.**

By using this matrix transformation to balance the Reducers, each Reducer will process either *c* pairs or *f* pairs of data blocks, where in theory all Reducers load are fully balanced and each Reducer only calculate about half of the pairs in an original Reducer. In the example shown in Figure 2, six pairs of data blocks are calculated with an imbalanced workload. After balancing using the above matrix transformation, we can see in Figure 4 each Reducer now only calculates two pairs of data block.

**Figure 5:**
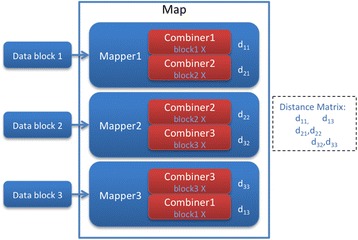
**Calculation in Combiner.** This is the MapReduce framework where all calculations in Reducers are moved to Combiner according to the algorithm in Figure 4.

Finally we have designed our workflow to take into account uneven numbers of available Reducers to Mappers. The hash function in MapReduce cannot always map Mappers’ output to the expected number of Reducers even if the Mapper output keys are well designed. For example, if six Mapper output keys are sent to six respective Reducers and only three Reducers are available to receive the data, this results in one or more Reducer receiving a greater workload to process sequentially. To ensure all Reducers are fully utilized, calculations in Reducers are shifted to Combiners, which read pairwise data blocks directly from the file system, that calculate the result at the Mapper stage before the hash mapping, as shown in Figure 5.

## Results and discussion

Our optimisations were tested against large datasets to validate being able to handle large studies that we would expect to see in tranSMART. We used three publicly available datasets: ONCOLOGY (GSE2109) [22] taken from NCBI GEO consisting on 2158 subjects and 54,675 probesets (1.9 GB comma-delimited value file), LEukemia (GSE13159) [23,24] consisting on 2096 subjects and 54,675 probesets (1.8 GB comma-delimited value file) MULTMYEL consisting on 559 subjects and 54,675 probesets (493 MB comma-delimited value file), and a breast invasive carcinoma dataset taken from TCGA [25] consisting of 547 subjects and 17,814 probesets (157 MB comma-delimited value file). We used IC Cloud [26], a computational testbed based at Imperial College London to set up comparable virtual machine configurations for the R implementation and our MapReduce implementation.

In order to verify the universality of our new method we tested all types of correlation functions. Currently, there are three main types of correlation coefficient calculation algorithms, product–moment coefficient, rank correlation coefficients and other dependence measurements. We took Pearson correlation for product–moment type, Spearman and Kendall for rank type and Euclidean distance for other dependence measures, which are implemented in R packages, r-base and fields. We compared a vanilla R instance and parallel R (Snowfall) against MapReduce via RHIPE.

Two benchmarks are used for the performance evaluation. The micro-benchmark used datasets MULTMYEL (559 subjects). The vanilla R configuration used 32 CPU cores and 32GB of memory in a single VM. Snowfall used 4 VMs each with 8 CPU cores, 8GB of memory and 6 workers. RHIPE used 4 VMs each with 8 CPU cores, 8GB of memory and 6 Mappers. The macro-benchmark used datasets ONCOLOGY, LEukemia and TCGA. Three main tests are performed using three datasets, including ONCOLOGY (2158 subjects), a cross-study consisting of ONCOLOGY and LEukemia (4254 subjects), and an artificial dataset consisting of dual ONCOLOGY and dual LEukemia (8508 subjects). Due to the extremely long execution time of Kendall correlation, only the smallest TCGA data was used to calculate Kendall correlation. The vanilla R configuration used 64 CPU cores and 64GB of memory in a single VM. The master node of Snowfall used 8 CPU cores, 8GB of memory and 6 workers, with 14 slave VMs each with 4 CPU cores, 4 GB of memory and 3 workers. The master node of RHIPE used 8 CPU cores and 8GB of memory using 6 Mappers, with 14 slave VMs each with 4 CPU cores and 4 GB of memory using 42 Mappers. Each experiment consists of two stages: data preparation and calculation. There are five methods for comparison: vanilla R, default Snowfall using socket connections, optimised Snowfall using the Rmpi [16] package, and RHIPE using both the basic MapReduce algorithm and the optimised one. The vanilla R data preparation is loading comma-separated value (CSV) data matrix from an Ext4 (fourth extended filesystem) local file system to an R matrix object. With default Snowfall, the data preparation overhead consists of loading the CSV data matrix from an Ext4 local file system, initializing the worker nodes, and exporting the data matrix and code to the workers. The data matrix is split by row (subject) rather than data block, where the corresponding computation calculates the correlation between rows. With Snowfall using Rmpi, the data preparation overhead includes splitting the data matrix using the Linux split command and copying each of the data block files to every VM. The number of data blocks depends on the number of workers. During the calculations, Rmpi workers perform the same tasks as in the Mappers in MapReduce. Each worker loads each corresponding data block sequentially from the local Ext4 filesystem. After each data block is loaded, the worker performs the corresponding calculations. Using RHIPE, the data preparation overhead consists of splitting the data matrix and uploading each file to HDFS [14]. The calculation then follows the algorithms described in the method section.

We carried out a performance evaluation between vanilla R, parallel R with Snowfall, and MapReduce implementation. We calculated a subject correlation on the all subjects, calculating the coefficient matrices of the two benchmarks using Euclidean distance, Pearson and Spearman correlation functions.

**Figure 6:**
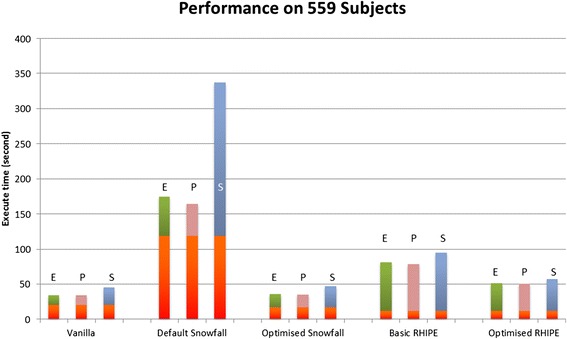
**Performance on the micro-benchmark.** This is the performance evaluation using vanilla R, default Snowfall package with socket connection, optimised Snowfall with Rmpi package and RHIPE package with the basic MapReduce algorithm and our optimised one. The bottom three bars in each method shows the data preparation time. The vanilla R data preparation indicates loading data from a local file into memory; while in all parallel R methods, data copy almost occupy the whole data preparation time. The upper three bars respectively indicate the Euclidean (E), Pearson (P) and Spearman (S) calculation time.

In the micro-benchmark, as shown in Figures 6, vanilla R performs fastest and default Snowfall performs the slowest. Vanilla R has a larger data preparation overhead than RHIPE, but the calculation itself greatly outperforms all the other methods. All parallel R methods do not perform any better due to the data communication overhead. There is an extreme example in default Snowfall Spearman where the parallel calculation is 9 times slower than vanilla R. The optimised RHIPE demonstrates a 3.26-5.83 fold increase compared to the default Snowfall. The optimised RHIPE conducts 1.56-1.64 times faster than the basic RHIPE, which almost achieves the expected two times acceleration, considering all the overheads, such as data preparation and Hadoop job initialization.

Though the optimised RHIPE is outperformed by optimised Snowfall, it has a lower data preparation overhead. This is advantageous as RHIPE is likely to be able to perform better overall with much larger datasets. Thus, we utilized the macro-benchmark to further test the optimised Snowfall and the optimised RHIPE with vanilla R as a baseline.

**Figure 7:**
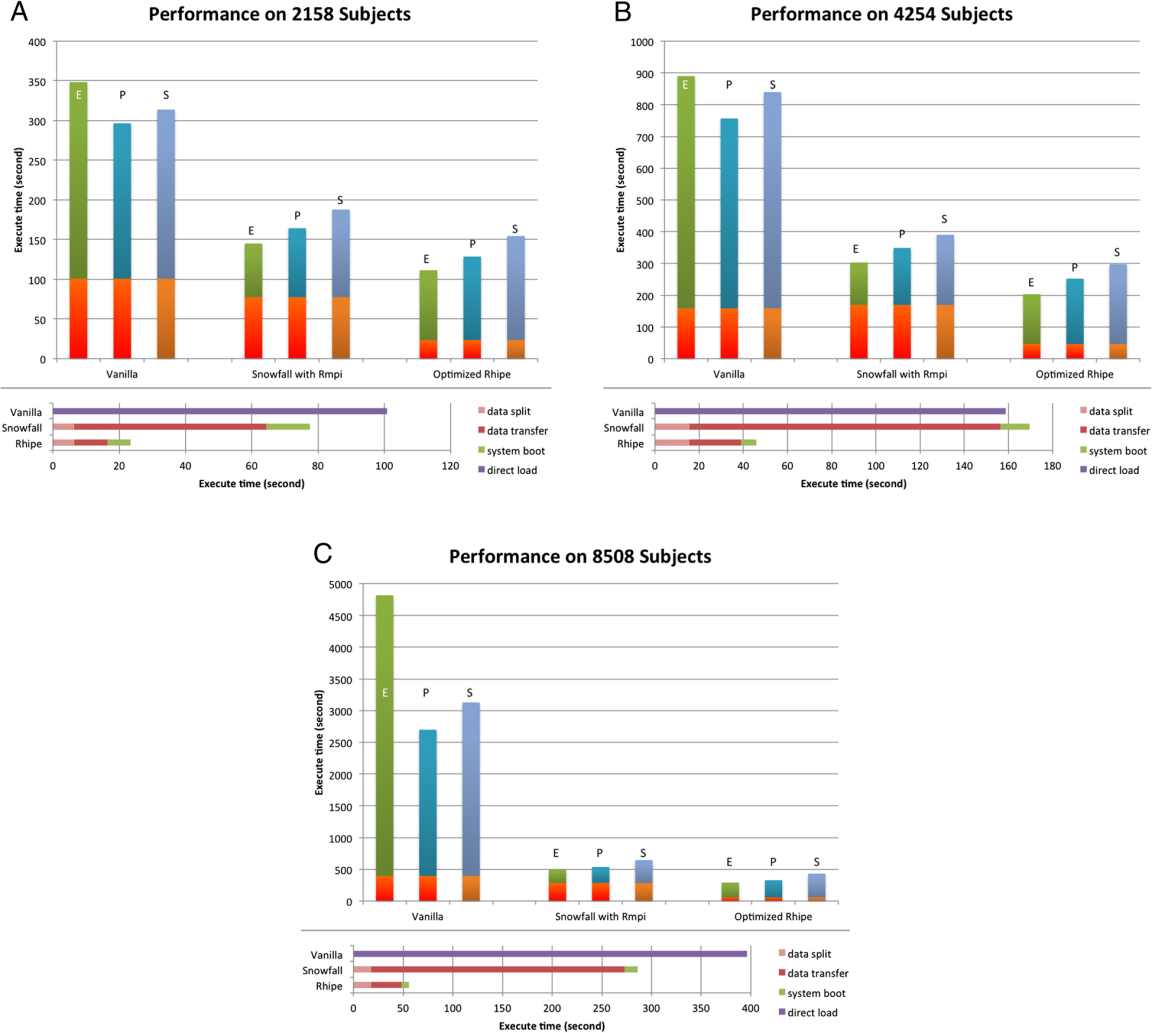
**Performance on the macro-benchmark.** This is the performance evaluation using vanilla R, optimised Snowfall and optimised RHIPE package. The upper part of each figure indicates the total execution time. In this part, the bottom three bars in each method shows the data preparation time; while the upper three bars respectively indicate the Euclidean (E), Pearson (P) and Spearman (S) calculation time. The lower part of each figure details the data preparation of each method. In this part, data split shows the time used for splitting the large data matrix into smaller pieces, data transfer for the Snowfall shows data copy time for the pieces to corresponding MPI workers, data transfer for the RHIPE shows the data uploading time for the same pieces to HDFS, system boot respectively shows the boot time of the MPI cluster and the Hadoop cluster, and the direct load shows the data loading time for vanilla R. **A**: Performance on ONCOLOGY dataset. (2158 subjects). **B**: Performance on the cross-study consisting of ONCOLOGY and LEukemia (4254 subjects). **C**: Performance on the large artificial dataset (8508 subjects).

In the tests using the macro-benchmark, as shown in Figure 7, the optimised RHIPE outperforms all other methods. Though the optimised RHIPE calculation time is still a little longer than optimised Snowfall, the optimised RHIPE outperforms the optimised Snowfall due to faster data transfer via HDFS and thus shorter data preparation times. In Figure 7A (2158 subjects), benefiting from the 3.30 times faster data preparation, the optimised RHIPE performs 1.22 - 1.30 times faster than the optimised Snowfall. In Figure 7B (4254 subjects), benefiting from the 3.69 times faster data preparation, the optimised RHIPE performs 1.31 - 1.49 times faster than the optimised Snowfall. In Figure 7C (8508 subjects), benefiting from the 5.13 times faster data preparation, the optimised RHIPE performs 1.50 - 1.71 times faster than the optimised Snowfall and 7.24-16.56 times faster than the vanilla R. We propose that RHIPE holds great promise for large data analysis with the data size increasing.

**Figure 8:**
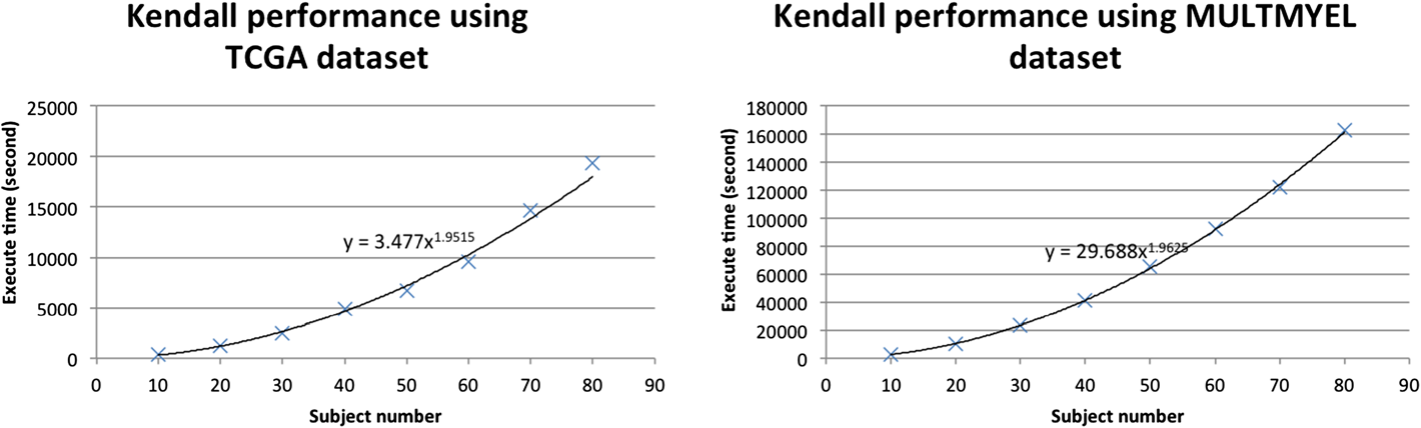
**Estimation of Kendall in vanilla R environment.**

As part of our baselines for comparison, we performed a full Kendall correlation calculation in our vanilla R configuration, where we found that the total execution time was indeterminately long. We used the TCGA and MULTMYEL datasets to estimate the full time because this scaling property of this particular dataset allows us to extrapolate the total calculation time more quickly. Each vector is a subject with about 54,675 probeset values. We started from 10 subjects to 80 subjects to simulate the trend and formula, as shown in Figure 8. We calculated, based on the observed trends, that for the processing all of the TCGA subjects the estimated execution time would be 759,668 seconds (about 9 days) and for MULTMYEL the estimated time would be 7,317,734 seconds (about 85 days).

We successfully performed the parallel Kendall correlation with all subjects of TCGA using RHIPE and Snowfall. The total execution time of the optimised RHIPE (24,939.31 seconds) is very similar to the optimised Snowfall execution time (24,607.29 seconds). Both of the data preparation times, less than 20 second, can be ignored comparing to the extremely long execution times. Both of these parallel methods perform approximate 30 times faster than vanilla R. The same parallel algorithms could be applied to the MULTMYEL and ONCOLOGY datasets. This test indicates the optimised RHIPE gradually downgrades to the optimised Snowfall in the tests with smaller input dataset but longer calculation time.

## Conclusions

In this paper, our work is aimed at creating an efficient data distribution and parallel calculation algorithm based on MapReduce to optimise the correlation calculation. We evaluate the performance of our algorithm using two gene expression benchmarks. In the micro-benchmark, our implementation using MapReduce, based on the R package RHIPE, demonstrates a 3.26-5.83 fold increase compared to the default Snowfall and 1.56-1.64 fold increase compared to the basic RHIPE MapReduce in the Euclidean, Pearson and Spearman correlations. In the macro-benchmark, with 3.30-5.13 times faster data preparation operations, the optimised RHIPE performs 1.22-1.71 times faster than the optimised Snowfall and 2.03-16.56 times faster than the vanilla R. Both the optimised RHIPE and the optimised Snowfall finish the long parallel Kendall correlation with all subjects of TCGA within 7 hours. Both of them conduct about 30 times faster than the estimated vanilla R. We propose that MapReduce framework holds great promise for large molecular data analysis, in particular for high-dimensional genomic data.
